# *Penicillium janthinellum* NCIM1366 shows improved biomass hydrolysis and a larger number of CAZymes with higher induction levels over *Trichoderma reesei* RUT-C30

**DOI:** 10.1186/s13068-020-01830-9

**Published:** 2020-12-01

**Authors:** AthiraRaj Sreeja-Raju, Meera Christopher, Prajeesh Kooloth-Valappil, Rajasree Kuni-Parambil, Digambar Vittal Gokhale, Meena Sankar, Amith Abraham, Ashok Pandey, Rajeev K. Sukumaran

**Affiliations:** 1 Biofuels and Biorefineries Section, Microbial Processes and Technology Division, CSIR-National Institute for Interdisciplinary Science and Technology, Industrial Estate, Pappanamcode, Thiruvananthapuram, 695019 India; 2 Academy of Scientific and Innovative Research (AcSIR), Ghaziabad, 201002 India; 3 Department of Microbiology and Cell Biology, Indian Institute of Science, Bangalore, 560012 India; 4 National Chemical Laboratory, Pune, India; 5 Department of Chemical Engineering, Hanyang University, Seoul, Republic of Korea; 6 Centre for Innovation and Translational Research, CSIR-Indian Institute of Toxicology Research, Lucknow, India

**Keywords:** *Penicillium janthinellum*, *Trichoderma reesei*, Cellulase, CAZymes, Secretome, Bioethanol

## Abstract

**Background:**

Major cost of bioethanol is attributed to enzymes employed in biomass hydrolysis. Biomass hydrolyzing enzymes are predominantly produced from the hyper-cellulolytic mutant filamentous fungus *Trichoderma reesei* RUT-C30. Several decades of research have failed to provide an industrial grade organism other than *T. reesei*, capable of producing higher titers of an effective synergistic biomass hydrolyzing enzyme cocktail*. Penicillium janthinellum* NCIM1366 was reported as a cellulase hyper producer and a potential alternative to *T. reesei*, but a comparison of their hydrolytic performance was seldom attempted.

**Results:**

Hydrolysis of acid or alkali-pretreated rice straw using cellulase enzyme preparations from *P. janthinellum* and *T. reesei* indicated 37 and 43% higher glucose release, respectively, with *P. janthinellum* enzymes. A comparison of these fungi with respect to their secreted enzymes indicated that the crude enzyme preparation from *P. janthinellum* showed 28% higher overall cellulase activity. It also had an exceptional tenfold higher beta-glucosidase activity compared to that of *T. reesei*, leading to a lower cellobiose accumulation and thus alleviating the feedback inhibition. *P. janthinellum* secreted more number of proteins to the extracellular medium whose total concentration was 1.8-fold higher than *T. reesei*. Secretome analyses of the two fungi revealed higher number of CAZymes and a higher relative abundance of cellulases upon cellulose induction in the fungus.

**Conclusions:**

The results revealed the ability of *P. janthinellum* for efficient biomass degradation through hyper cellulase production, and it outperformed the established industrial cellulase producer *T. reesei* in the hydrolysis experiments. A higher level of induction, larger number of secreted CAZymes and a high relative proportion of BGL to cellulases indicate the possible reasons for its performance advantage in biomass hydrolysis.

## Background

Lignocellulosic biomass is mainly composed of three polymers—cellulose, hemicellulose, and lignin. Among them, cellulose is the most abundant component which consists of 35–50% of plant dry weight followed by hemicellulose (20–35%) and then lignin (5–30%) [1]. Degradation of lignocelluloses is generally carried out by a set of enzymes including ligninases, hemicellulases, cellulases and other accessory enzymes. Their relative proportions and quantities can determine the efficiency of hydrolysis [2]. The major cost involved in bioethanol production from lignocellulosic biomass is contributed by the hydrolysis step, owing to the cost of production of these enzymes [3]. Thus, reducing the cost of enzymes is critical for making the process economical. The major sources of lignocellulose-degrading enzymes are filamentous fungi, mainly the genera *Trichoderma*, *Aspergillus* and *Penicillium* [4]. With cellulose being the major component, cellulases play an important role in the degradation process. Cellulases are mainly divided into three major groups based on their mode of action on cellulose: (1) endoglucanases, which randomly cleave internal β-1, 4 linkages in cellulose chain generating free ends; (2) cellobiohydrolases, which act in a processive manner on either reducing or non-reducing ends of the cellulose chain, releasing cellobiose as the major product and (3) beta-glucosidases that hydrolyze cellobiose into glucose [5].

Among the filamentous fungi, *Trichoderma reesei* is the most studied cellulase producer and considered as the model organism for cellulase research. It was originally isolated from the Solomon Islands during the Second World War, and the isolate was named *T. reesei* QM6a as part of the culture collection at the US Army Quarter Master Research and Development Center at Natick, Massachusetts. Attempts to improve the cellulase production from *T. reesei* QM6a by several generations of mutagenesis resulted in an enzyme hyper-secreting mutant named as *T. reesei* RUT-C30 [6]. Currently, *T. reesei* RUT-C30 is the predominantly used industrial cellulase producer, because of its ability to produce a high titer of cellulases and a gene regulation mechanism that is highly adapted for cellulose utilization [7]. Nevertheless, the cost of cellulases is still a major concern, with only a handful of companies commercially manufacturing biomass hydrolysis enzymes, and transportation and storage of the enzyme itself adding significantly to the cost [8]. Availability of an alternate cellulase source, better than *T. reesei* in terms of higher yields, an effective ratio of different glucanases to effect improved biomass hydrolysis performance, and/or better production economics as determined by higher specific activities, shorter fermentation times, etc., would be highly advantageous to the 2G-ethanol industry, especially in countries where commercial production of efficient biomass hydrolyzing cellulases are not available.

*Penicillium janthinellum* NCIM 1171 is a filamentous soil fungus known for its efficient cellulase production and hydrolysis efficiency [9, 10]. Classical mutagenesis studies conducted at CSIR-NCL, Pune had yielded three mutants of *P. janthinellum* NCIM 1171 with enhanced cellulase production and they were named as EMS-UV-8 (NCIM1366), EU-21 and EU2D-21 [11–13]. The mutant EMS-UV-8, named as isolate NCIM 1366 was reported to have higher beta-glucosidase (BGL) activity compared to other mutants [11]. While it’s enzyme was not the best in terms of hydrolyzing pure cellulose [11], it was efficient in the hydrolysis of pretreated rice straw (unpublished results).The strain was also successfully used for bioethanol production from pretreated wheat straw [14]. Since the mutant strain produced cellulases efficient in hydrolysis of natural biomass substrates, *P. janthinellum* NCIM1366 was chosen as the model strain to be compared with *T. reesei* RUT-C30, the best known industrial producer of cellulase. Secretome analyses performed using liquid chromatography tandem mass spectrometry (LC–MS/MS) revealed higher number of CAZymes in *P. janthinellum* compared to *T. reesei,* and a higher relative abundance of cellulases upon induction using cellulose, which may explain the higher activity and better biomass hydrolytic performance of enzyme preparation from the fungus.

## Results

### *P. janthinellum *secretes higher amount of proteins compared to *T. reesei*

**Fig. 1:**
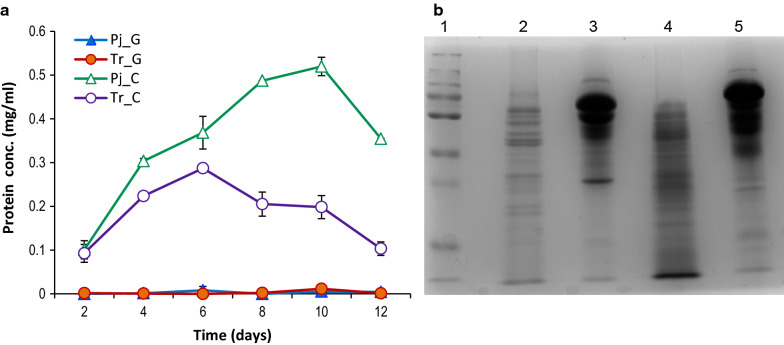
Extracellular protein production by *T. reesei* and *P. janthinellum* in response to induction by cellulose. **a** Extracellular protein concentrations in *T. reesei* (Tr) and *P. janthinellum* (Pj) at different time points when cultivated in glucose and in cellulose. **b** SDS-PAGE showing differences in protein production by the two fungi. Lane information—1) Page Ruler ® pre-stained protein ladder, 2) Tr grown in glucose 3) Tr. grown in cellulose as sole C source 4) Pj grown in glucose 5) Pj. grown in cellulose as sole C source

Cellulose is a large polymer and utilization of it requires secretion of enzyme by microorganisms to process it outside the cell, so that the simple sugars derived from its breakdown can be taken inside. The total secreted protein concentration in presence of cellulose is indicative of the efficiency of the fungus in utilizing the polymer, as efficient cellulose digestion typically requires a milieu of different enzyme activities, in addition to cellulases. *T. reesei* showed a maximum protein secretion of 0.28 mg/ml on the 6th day of growth, whereas *P. janthinellum* secreted the maximum protein on 10th day of growth which was ~ 1.8 times higher than *T. reesei* (Fig. 1a). At all the time points tested, extracellular protein concentration was higher in *P. janthinellum*. SDS-PAGE of the extracellular fractions from both fungi under uninduced (glucose grown) and induced (cellulose grown) conditions indicated significant elevation in secreted proteins upon cellulose induction (Fig. 1b). It was also observed that visibly, a greater number of extracellular proteins were secreted by *P. janthinellum.*

### *P. janthinellum* shows lesser cellobiose accumulation in the hydrolysis medium which is indicative of its better beta-glucosidase activity

**Fig. 2:**
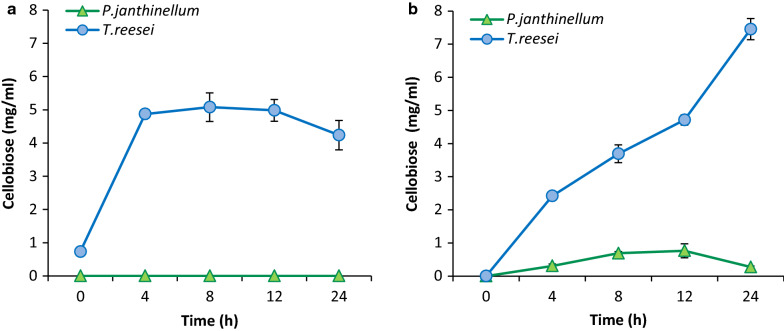
Cellobiose accumulation during hydrolysis of biomass using *T. reesei* or *P. janthinellum* enzymes. **a** Acid-pretreated rice straw, **b** alkali-pretreated rice straw

Cellobiose, the intermediate product of enzymatic cellulose hydrolysis, is produced through action of exoglucanases and is the substrate for cellobiase/beta-glucosidase. Cellobiose accumulation can lead to product inhibition of upstream enzymes (endoglucanases and cellobiohydrolases) thus slowing down the whole hydrolytic process [15, 16]. *T. reesei* is known to have limited cellobiase/beta-glucosidase (BGL) activity, and while it may be advantageous for the organism in tight regulation of cellulose metabolism while growing on natural substrates, it is a disadvantage for the biomass hydrolyzing enzyme cocktails produced using the fungus and often the *T. reesei* enzyme’s lack of BGL activity is compensated by addition of BGL enzyme from other organisms. In this study, it was observed that the cellobiose accumulation in the hydrolysis mixtures was higher for the *T. reesei* enzyme compared to *P. janthinellum,* indicating an incomplete digestion due to the inherent low BGL activity of the former (Fig. 2). For acid-pretreated rice straw, there was little or no cellobiose accumulation observed during the 24 h of hydrolysis, in the case of *P. janthinellum* enzyme, while about 5 mg/ml cellobiose was observed consistently from 4th hour onwards in the case of *T. reesei*. In alkali pretreated rice straw hydrolysis, the cellobiose concentration increased from 2.42 mg/ml in 4 h to 7.45 mg/ml in 24 h for *T. reesei* while the maximum cellobiose accumulation in the case of *P. janthinellum* was only 0.76 mg/ml at 12 h after which it again decreased. This indicated the efficient removal of cellobiose, from the reaction medium by *P. janthinellum* enzyme, which could be accounted for by the almost tenfold higher beta-glucosidase activity in the fungus. The results are an indicative of an optimum enzyme cocktail from a single fungus that outperforms the conventional cellulase producer.

### Cellulases from *P. janthinellum *perform better than *T. reesei* cellulases in the hydrolysis of pretreated biomass

**Fig. 3:**
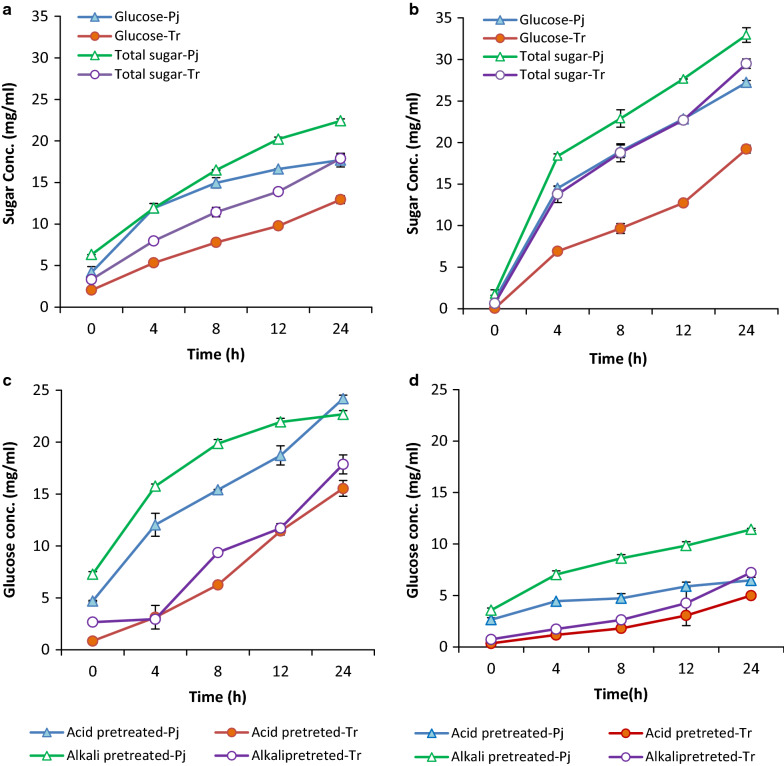
Sugar release on enzymatic hydrolysis of pretreated biomass by cellulases from *T. reesei* RUT C30 and *P. janthinellum* NCIM 1366. Glucose and total sugar release on enzymatic hydrolysis using *T. reesei* (Tr) and *P. janthinellum* (Pj) cellulases from **a** acid-pretreated rice straw and **b** alkali-pretreated rice straw. Glucose release on hydrolysis using Tr or Pj cellulases from **c** sugarcane bagasse, **d** eucalyptus leaves

Both the dilute acid and dilute alkali pretreated rice straw were hydrolyzed better by *P. janthinellum* cellulases compared to enzymes from *T. reesei,* indicated by a significantly higher glucose release (Fig. 3a and b). At 24 h, glucose release by *T. reesei* and *P. janthinellum* cellulases from acid-pretreated rice straw were 12.94 ± 0.8 mg/ml and 17.69 ± 0.47 mg/ml, respectively, the latter showing a 37% higher glucose release. Similar results were observed for alkali-pretreated rice straw, where *P. janthinellum* enzyme released 27.24 ± 0.22 mg/ml of glucose which was 43% higher than the *T. reesei* cellulase. Also, the glucose release for both the acid and alkali pretreated biomasses was higher with *P. janthinellum* cellulase at all the measured time points. Total sugar release from acid-pretreated rice straw, calculated as the sum of the concentrations of glucose, xylose, arabinose and mannose in the hydrolysis mixture, was 17.88 ± 0.5 mg/ml for *T. reesei* enzyme and 22.41 ± 0.23 mg/ml for *P. janthinellum* enzyme. For alkali-pretreated rice straw, the hydrolysate sugar contents were 29.49 ± 0.57 mg/ml and 32.94 ± 0.87 mg/ml, respectively, for *T. reesei* and *P. janthinellum* enzymes (Fig. 3a and b). *P. janthinellum* enzyme released 25% higher glucose and 11% higher total sugars from alkali pretreated rice straw sugars compared to *T. reesei* enzyme.

The choice of substrate for the hydrolysis reaction often affects the efficiency of glucose release, and enzyme performance could be different on different biomass substrates. Two different biomasses with significantly different properties, viz. sugar cane bagasse and Eucalyptus leaves pretreated using acid or alkali, were used as substrates for testing hydrolytic efficiency of enzymes from both the fungi. Both the biomasses, regardless of the method of pretreatment were hydrolyzed better by *P. janthinellum* enzyme, indicated by higher glucose release. For acid-pretreated sugarcane bagasse, the glucose release at 24 h was 24.19 ± 0.34 for *P. janthinellum* enzyme and 15.54 ± 0.76 for *T. reesei* enzyme, while in the case of alkali-pretreated biomass, it was 22.16 ± 0.35 and 17.87 ± 0.91, respectively, for *P. janthinellum* and *T. reesei*. Similar results were obtained for eucalyptus leaves, for which the glucose release across treatments were less compared to other biomass types (Fig. 3c and d).

### *P. janthinellum* produces higher enzyme titers compared to *T. reesei*

**Fig. 4:**
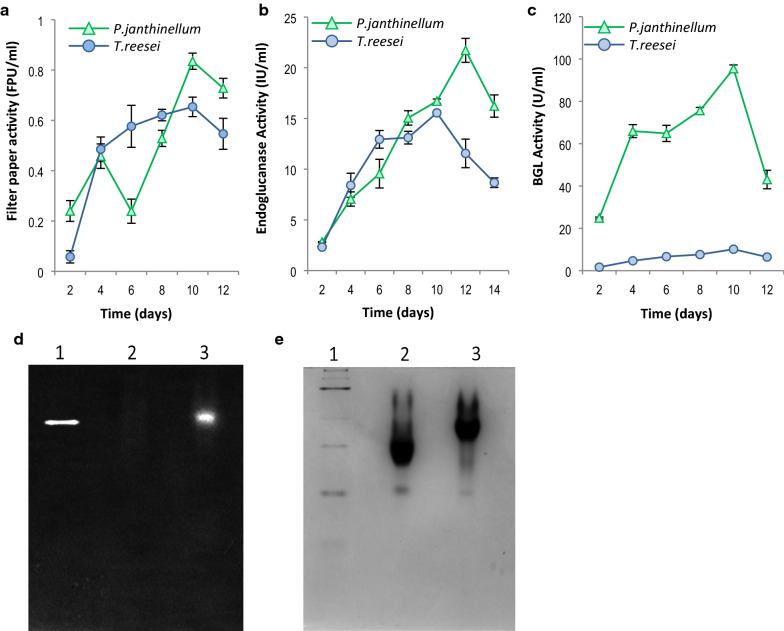
Extracellular cellulase production by *T. reesei* and *P. janthinellum* in Mandels and Weber medium with cellulose as sole carbon source. **a** Total cellulase activity (filter paper units), **b** endoglucanase activity (CMCase), **c** beta-glucosidase (BGL) activity. **d** Zymogram analysis showing BGL activity as MUG fluorescence. **e** Native PAGE showing protein profile of *T. reesei* and *P. janthinellum* secreted enzymes (Lane1—NativeMark™ unstained protein standard from Invitrogen, Lane2—*T. reesei* enzyme extract, Lane3—*P. janthinellum* enzyme extract for both **d** and **e**

To know how each of the major components of cellulolytic system contribute to the hydrolytic efficiency of *P. janthinellum* cellulase cocktail, standard cellulase assays were performed, on the enzymes produced by the fungi. Extracellular enzyme production in this case was carried out using the same medium and under identical conditions of growth. Secreted enzymes from both fungi were analyzed for the total cellulase, endoglucanase and beta-glucosidase activities. Both *T. reesei* and *P. janthinellum* showed maximum cellulase activity on the 10th day, but the FPAse activity of *P. janthinellum* (0.83 FPU/ml) was 28% higher than that of *T. reesei* (0.65 FPU/ml) (Fig. 4a). Peak endoglucanase activity of 21.72 IU/ml was shown by *P. janthinellum* on the 12th day, whereas *T. reesei* showed maximum activity (15.55 IU/ml) at 10th day (Fig. 4b). *T. reesei* showed an endoglucanase activity of 12.93 IU/ml even at 6th day, but the levels were raised only upto 15.55 IU/ml on 10th day and were not sustained at further time points, probably indicating a feedback inhibition through glucose accumulation. *P. janthinellum* on the contrary, had a lower initial endoglucanase activity (9.55 IU/ml) which steadily increased to 21.72 IU/ml on 12th day and showed an ascending trend. The largest difference in enzyme activity between the two fungi was observed in the case of beta-glucosidase (BGL) activity. Highest BGL activity in the case of *T. reesei* was 10.15 U/ml. *P. janthinellum* also showed the highest BGL activity (95.42 U/ml) on the 10th day (Fig. 4c). Also, the fungus produced 24.88 U/ml activity on the 2nd day, where *T. reesei* could elaborate only 1.68 U/ml. These results are remarkable as the beta-glucosidase activity at peak levels by the two fungi is different by an almost tenfold margin. Also, it becomes evident that the expression of BGL sets in early in the *P. janthinellum* which would allow it to hydrolyze cellulose faster and prevent cellobiose accumulation, which in turn may help to overcome an early setting in of feedback inhibition. The results were also confirmed by a zymogram analysis which showed a prominent BGL activity band in *P. janthinellum*, whereas the *T. reesei* BGL band was barely visible (Fig. 4d).

### Comparative secretome analysis of cellulose-induced cultures confirm secretion of a relatively larger number of CAZymes and lignocellulose active enzymes by *P. janthinellum*

**Fig. 5:**
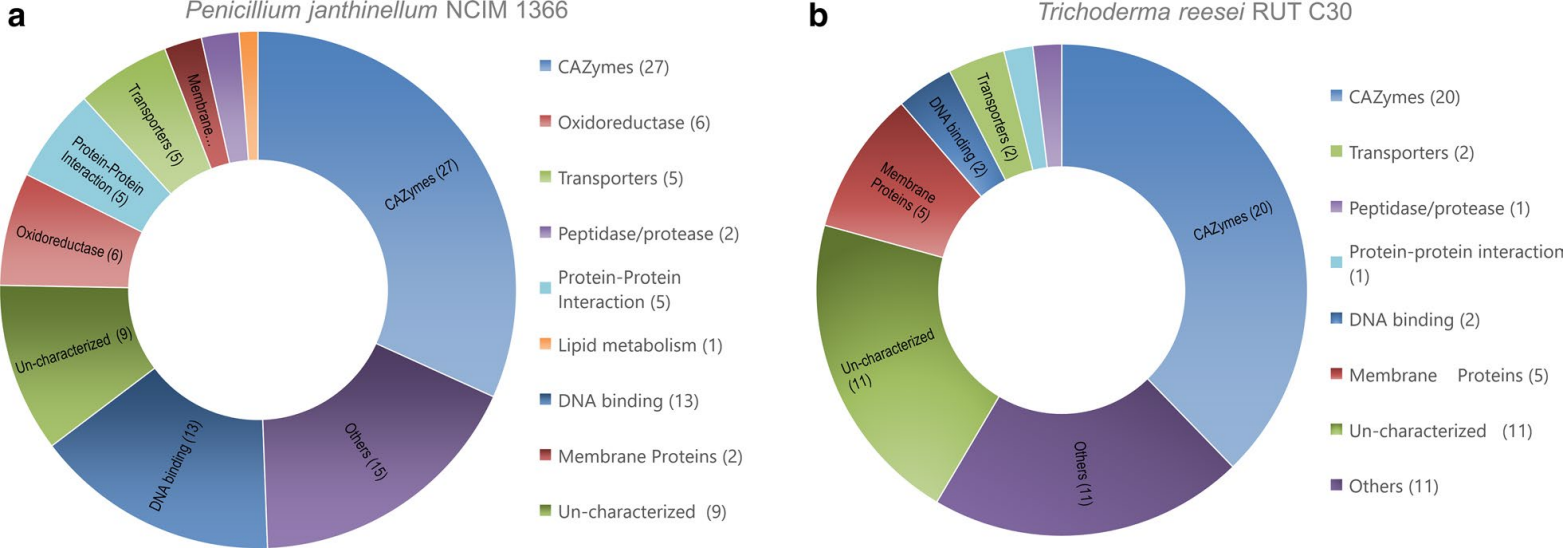
Functional categories of proteins secreted on cellulose induction by *P. janthinellum* and *T. reesei*. **a** *P. janthinellum*, **b** *T. reesei*

**Fig. 6:**
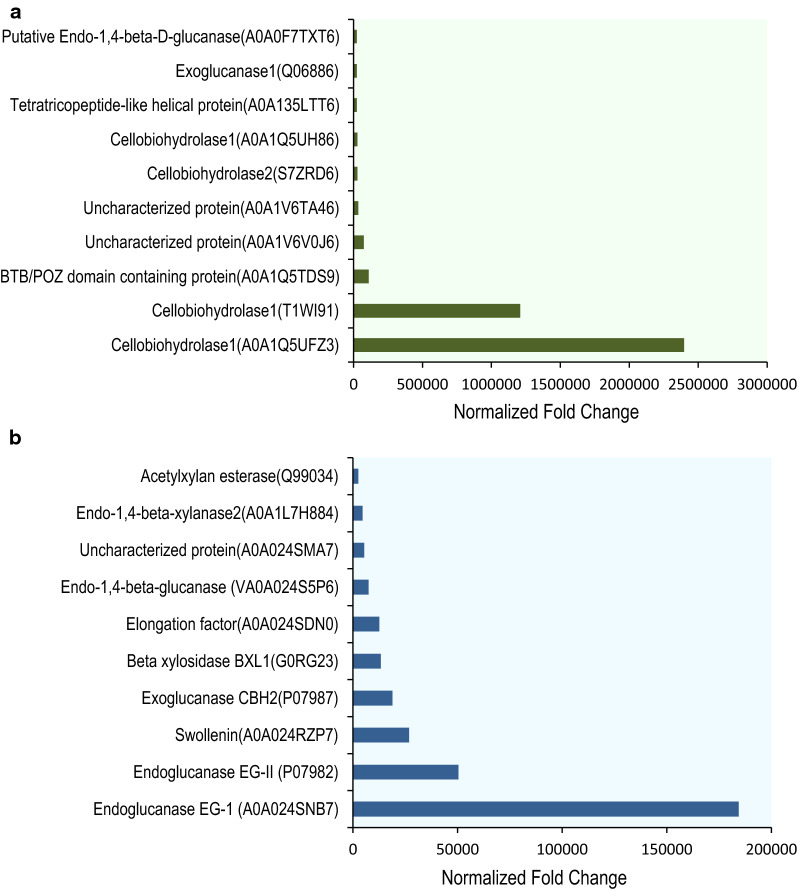
Most highly expressed proteins in the secretome of *P. janthinellum* and *T. reesei,* on cellulose induction. **a** *P. janthinellum*, **b** *T. reesei*

The observed cellulase activity and hydrolysis activity are contributed by the extracellular enzymes in both organisms. As both cultures showed maximum filter paper activity on 10th day of inoculation in cellulose medium, it was speculated that the maximum repertoire of enzymes are secreted at that time point. For comparison glucose was selected as the non-inducing carbon source. The secreted proteins from both cultures, either grown with glucose as carbon source or upon induction with cellulose on the 10th day of growth, were identified and quantitatively analyzed by liquid chromatography tandem mass spectrometry (LC–MS/MS) analysis. Additional file 1: Table S1, and Additional file 2: Table S2 lists all the proteins, their Uniprot accession number, molecular weights, number of unique peptides, normalized abundance in glucose and cellulose grown cultures and fold change of upon induction for both *T. reesei* RUT-C30 and *P. janthinellum* NCIM1366 cultures, respectively. Our analysis detected a total of 53 proteins from *T. reesei* and 85 proteins from *P. janthinellum* in the 10th day secretome. The distribution of proteins according to their biological function is shown in Fig. 5. Among them, 27 proteins from *T. reesei* (51%) and 29 proteins from *P. janthinellum* (34%) were predicted to have an N terminal signal peptide using SignalP5.0 server. The identification of proteins without a signal peptide in the secretome could be indicative of the presence of cell lysis, cell death, or secretion through unconventional mechanisms [17]. Figure 6 shows the top 10 highly expressed proteins, as measured by the normalized abundance of their peptides in cellulose-induced cultures, compared to the control (grown in glucose). Most of the highly expressed proteins from both organisms were directly involved in the lignocellulose degradation.

**Fig. 7:**
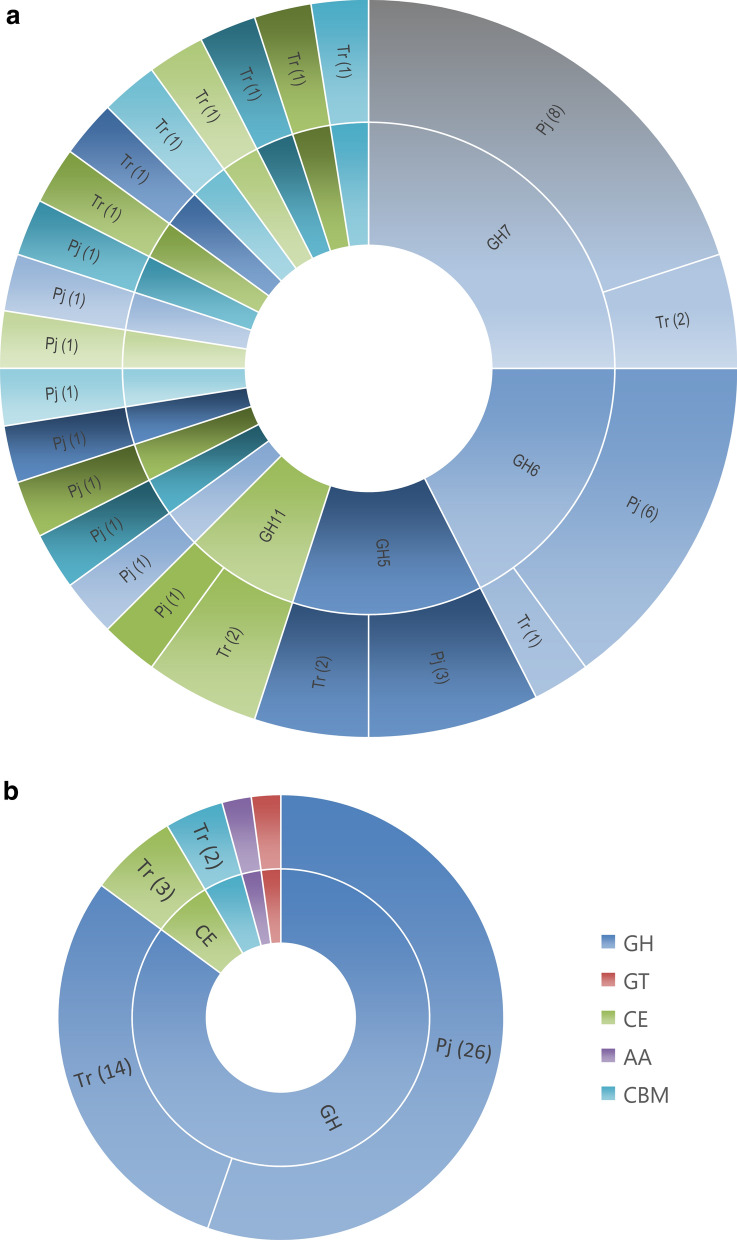
Distribution of secreted proteins of the fungi in different CAZyme families, and the glycosyl hydrolase family representation in the secretome. **a** Secreted protein distribution in CAZyme families, **b** distribution of glycosyl hydrolases in subfamilies. Tr, *T. reesei;* Pj, *P. janthinellum*

Since it appeared from the forgoing studies that the reason for better overall cellulolytic activity and hydrolytic efficiency of *P. janthinellum* could be its secretion of a larger number of proteins, most of which are known to be involved in lignocellulose hydrolysis, it was speculated that the organism could elaborate more cellulolytic enzymes and/or accessory proteins compared to the industrial workhorse—*T. reesei*. An analysis of the CAZymes in the total secretomes was performed to understand their distribution in the extracellular proteins of both fungi. Among the 53 secreted proteins detected in *T. reesei*, 20 were identified as CAZymes (Fig. 5a) and the number of CAZymes identified in the 85 identified secreted proteins of *P. Janthinellum* were 27 (Fig. 5b). The distribution of proteins among different CAZy families and the distribution of glycosyl hydrolase (GH) family proteins in both the fungi are shown in Fig. 7. CAZymes from *P. janthinellum* were mostly GH family proteins except one in GT family. The CAZymes from *T. reesei* RUT-C30 were distributed to more CAZy families which included GH (glycoside hydrolases), CE (carbohydrate esterases), AA (auxiliary activities) and CBM (carbohydrate-binding module). In the case of GH family proteins, *P. janthinellum* secretome had almost double the number of different Glycoside Hydrolases compared to *T. reesei*. GH family proteins from *P. janthinellum* spanned over 12 GH subfamilies while for *T. reesei* it was 11 GH subfamilies. GH subfamilies 5, 6, 7 and 11 were detected in both secretomes while GH subfamilies 3, 4, 16, 17, 30 and 72 were detected only in *T. reesei* and GH subfamilies 2, 15, 27, 28, 36, 43, 55 and 75, were detected only in *P. janthinellum*.

Table 1 shows the list of CAZymes identified from the secretomes of *P. janthinellum* and *T. reesei*. Among the CAZymes detected, a total of 17 enzymes which are directly involved in cellulose hydrolysis were detected, of which 3 were common to both fungi, which were cellobiohydrolase1 (CBH1) (Uniprot accession: P62694, A0A088DLG0), cellobiohydrolase2 (CBH2) (P07987, F1CHI2) and endoglucanase 1 (EG-1) (A0A024SNB7, A0A0F7TSC9). The two cellobiohydrolases showed higher abundance in *P. janthinellum* and the endoglucanase showed higher abundance in *T. reesei*. Apart from the EG-1, two other endoglucanases, EG-II (P07982) and EG-V (A0A024S5P6), are identified from *T. reesei.* In *P. janthinellum*, 10 cellobiohydrolases and 2 endoglucanases were identified from the common cellulases. However, it may be noted that multiple peptide tags may be matching the same *P. janthinellum* NCIM 1366 gene sequence in reality, which may not be captured on analyzing against the genome (s) of other *Penicillium* species’ genomes as is the case here. While *P. janthinellum* exhibited 10 times the BGL activity of *T. reesei*, no beta-glucosidases were identified in both the fungi. Therefore, there was no way to confirm if the higher BGL activity obtained experimentally for *P. janthinellum* correlates to a higher amount of the corresponding protein in the secretome. It was previously observed that the BGL proteins had high specific activities and minute quantities can give high hydrolytic efficiencies, even though their proteins were undetectable by conventional means.

**Table 1:** CAZymes identified from the secretomes of *P. janthinellum* and *T. reesei*

CAZy family	Enzyme	Uniprot accession(s)	Tr-Glu	Tr-Cel	Tr-FC	Pj-Glu	Pj-Cel	Pj-FC	Presence
Cellulose hydrolysis/metabolism									
G H5	Endoglucanase EG-II	P07982	1.92	96905	50333	–	–	–	Tr
GH5	Endoglucanase B	A0A1Q5UIH7	–	–	–	14.5	1256	86	Pj
GH5	Putative endo-1,4-beta-D-glucanase	A0A0F7TXT6	–	–	–	0	22950	22950	Pj
GH6	Exoglucanase 6A	A0A1Q5UI44	–	–	–	0	11744	11744	Pj
GH6	Exoglucanase CBH2	P07987, F1CHI2	13.6	256595	18844	0.24	4989	20,734	Pj & Tr
GH6	Cellobiohydrolase	A0A1Q5UIA5	–	–	–	0.33	6916	20958	Pj
GH6	Cellobiohydrolase	A0A0F7TT91	–	–	–	–	–	–	Pj
GH6	Cellobiohydrolase	A0A1F5LUH2	–	–	–	–	–	–	Pj
GH7	Exoglucanase CBH1	P62694, A0A088DLG0	925	388093	419	0.13	2607	20054	Pj & Tr
GH7	Glucanase	S7ZRD6	–	–	–	0	28328	28328	Pj
GH7	Cellobiohydrolase1	A0A1Q5UFZ3	–	–	–	0.0036	8742	2396804	Pj
GH7	Cellobiohydrolase1	A0A1Q5UH86	–	–	–	0.36	10266	28516	Pj
GH7	Cellobiohydrolase1	T1WI91	–	–	–	0.00058	711	1225862	Pj
GH7	Exoglucanase1	Q06886	–	–	–	14.6	345207	23487	Pj
GH7	Cellobiohydrolase	A0A0F7TQ87	–	–	–	–	–	–	Pj
GH7	Endoglucanase EG-1	A0A024SNB7, A0A0F7TSC9	0.42	77136	183567	29.5	12605	426	Pj & Tr
GH45	Endo-1,4-beta-glucanase V	A0A024S5P6	3	22586	7407	–	–	–	Tr]
Hemicellulose hydrolysis/metabolism									
GH2	Beta-mannosidase	A0A2H3IHX4	–	–	–	11.8	240	20	Pj
GH3	Beta-xylosidase BXL1	G0RG23	2.8	37715	13278	–	–	–	Tr
GH5	Mannan endo-1_4-beta-mannosidase	Q99036, A0A1Q5SW99	12.8	1387	107	24.4	1891	77	Pj & Tr
GH11	Endo-1,4-beta-xylanase2	A0A1L7H884, A0A088S933	0	4530	4530	20	833	40	Pj & Tr
GH11	Endo-1,4-beta-xylanase1	A0A2H3A3Q5	110	32909	299	–	–	–	Tr
GH17	Beta-1,3-endoglucanase	A0A024SAF4	385	264	1.4	–	–	–	Tr
GH27	Alpha-galactosidase	A0A0F7KIJ2	–	–	–	0	5173	5173	Pj
GH30	Endo-beta-1,4-xylanase4	A0A024RV01	34.5	3052	88	–	–	–	Tr
GH43	Putative exo-beta-1,3-galactanase	S7ZCW4	–	–	–	78.2	1900	24	Pj
GH55	Exo-beta-1,3-glucanase	S8BDR6	–	–	–	117	13.9	8.4	Pj
CE05	Acetylxylan esterase	Q99034	19.5	49211	2522	–	–	–	Tr
CE16	Acetyl esterase	A0A024SFG8	10.8	24711	2273	–	–	–	Tr
CE16	Acetyl esterase	A0A2H3A8G3	18.6	1573	84	–	–	–	Tr
Chitin hydrolysis/metabolism									
GH18	Chitinase2	A0A024S0K1	11.2	597	53	–	–	–	Tr
GH75	Endo-chitosanase	A0A1Q5UKX2	–	–	–	422	1340	3.1	Pj
GH84	Putative bifunctional alpha-glucuronidase/N-acetyl beta-glucosaminidase	S7ZNU5	–	–	–	534	1572	2.9	Pj
Starch hydrolysis/metabolism									
GH15	Glucoamylase	A0A093V3D6	-	–	–	223	0	0.0044	Pj
Pectin									
GH28	Endo-polygalacturonase	A0A1Q5UPK8	–	–	–	56	3631	64	Pj
Accessory activities/other enzymes									
AA9	Polysaccharide monooxygenase CEL61A	A0A024SM10	2.85	2817	987	–	–	–	Tr
CBM1	Swollenin	A0A024RZP7	0.75	20249	26748	–	–	–	Tr
CBM1	CBM1 domain containing protein	A0A024SJK4	104	3661	35	–	–	–	Tr
GH16	Glucanosyl transferase	A0A024S7E6	787	2014	2	–	–	–	Tr
GH36	Putative galactinol sucrose galactosyl transferase	A0A1V6YI05	–	–	–	0	2804	2804	Pj
GH72	1_3-Beta-glucanosyl transferase	A0A024RXP9	7.9	3225	405	–	–	–	Tr
GT31	Glycosyl transferase	B8MG06	–	–	–	20	825	40	Pj

Tr, *Trichoderma reesei* RUT C30, Pj, *Penicillium janthinellum* NCIM 1366,

Tr-Cel, Pj-Cel, *T. reesei* or *P. janthinellum* grown with cellulose as carbon source (induced),

Tr-Glu, Pj-Glu, *T. reesei* or *P. janthinellum* grown with glucose as carbon source (uninduced)

There were 9 enzymes involved in hemicellulose degradation identified from the secretome of *T. reesei,* while 6 were identified in *P. janthinellum,* of which 2 were common with *T. reesei*. Chitin-degrading enzymes were also identified from both secretomes but pectin-degrading enzymes were identified only in *P. janthinellum*. Accessory activities known to aid cellulose hydrolysis in *T. reesei*, swollenin (A0A024RZP7) and lytic polysaccharide monooxygenase (A0A024SM10) were identified in the secretome of *T. reesei*, while these activities were not detected in *P. janthinellum*. In general, the relative abundance of most of the CAZymes were high in *P. janthinellum* compared to *T. reesei* and one of the cellobiohydrolases (A0A1Q5UFZ3) from GH7 family showed a very high relative abundance of 2.3 million. Though *P. janthinellum* did not show the accessory enzymes/activities in its secretome, it does not necessarily mean that the fungus lacks them, and further confirmations from the genome analysis is awaited.

## Discussion

Lignocellulose-degrading enzymes are critical in biomass conversion to biofuels and filamentous fungi are typically used for the production of these enzymes because of their ability to synthesize and secrete a wide array of plant cell wall-degrading enzymes [18]. *T. reesei* RUT-C30 is the most widely used fungus for cellulase production, despite it having a lower titer of beta-glucosidases and much lesser number of CAZymes compared to certain other fungi. This is primarily due to the fact that *T. reesei* produces the highest known titers of enzymes, the extracellular protein concentrations reaching as high 100 g/L [19]; and there is a wealth of information accumulated on its genetics and gene regulation through works spanning several decades [20]. However, there are still efforts targeted at improving its enzyme production [21, 22] as the cost of cellulases cannot yet be considered as economical for biorefinery operations. *P. janthinellum* NCIM 1366 is a mutant strain developed at the National Chemical Laboratory, Pune, India, through classical mutagenesis and which exhibited enhanced cellulase production compared to the parent strain NCIM 1171 [11]. The extracellular enzyme preparation from the strain was found in this study to be more efficient than the *T. reesei* enzyme in the hydrolysis of pretreated rice straw. The cellulase system of the fungus is relatively unexplored and the present study aimed to study the cellulases from *P. janthinellum* and compare it with established cellulase hyper producer *Trichoderma reesei* RUT-C30.

Experiments were designed to assess the efficiencies of both cellulase preparations for the hydrolysis of rice straw, pretreated using the most common methods of dilute acid or dilute alkali treatment at high temperature. The enzyme from *P. janthinellum* hydrolyzed the pretreated rice straw biomass better, indicated by the higher glucose release. Interestingly, the glucose release from acid and alkali pretreated rice straw was, respectively, 37% and 43% higher compared to *T. reesei*. Total sugar release was also higher for *P. janthinellum* enzyme extract. Similar to rice straw, *P. janthinellum* enzyme released higher amount of glucose from sugarcane bagasse and eucalyptus leaves. The results were surprising for a “new” cellulase producer to outperform the established industrial producer. Hence the extracellular enzyme preparations from both fungi were analyzed for their major component activities. These included endoglucanases (EGs), cellobiohydrolases (CBHs), and β-glucosidases (BGLs), which act in synergism to hydrolyze cellulose [23]. Since the parameters like media composition, pH, carbon source used, etc., can influence the quantity and variety of the cellulase components produced by fungi [24], the basic mineral salts medium of Mandels and Weber [25] with cellulose as the sole carbon source was used for both the organisms to obtain un-biased data. The results showed higher activity for all the three major components with peak activity on 10th day of incubation. Among the enzymes, the largest difference in activity was observed for beta-glucosidase (BGL), which was tenfold higher in *P. janthinellum.* It is already recognized that the extracellular enzymes of *T. reesei* strains are limited in BGL activity for effective biomass hydrolysis [26]. Thus it may be speculated that the higher BGL activity may be one of the significant factors which can contribute to the higher hydrolytic efficiency shown by *P. janthinellum* cellulase. This was also supported by the fact that the hydrolysis using *T. reesei* cellulase accumulated higher concentration of cellobiose in the medium, indicative of an incomplete hydrolysis. Thus, unlike *T. reesei* enzyme which has to be supplemented with external BGL for hydrolysis reaction [27], *P. janthinellum* enzyme preparations may be used without the need for any blending or with minimal addition of synergistic BGL preparation (s). In addition to the major cellulases, the total extracellular proteins were also 1.8 times higher for *P. janthinellum* and the qualitative analysis by SDS-PAGE showed more number of proteins in the gel complementing this finding.

Proteomic approaches have been widely used in filamentous fungi for the identification of both intracellular and extracellular proteins [28]. The genome of *T. reesei* QM6a, which is the parent strain of RUT-C30 was first sequenced in 2008 giving insight into its CAZyme system [29]. *T. reesei* is known to encode at least 10 cellulases, 16 hemicellulases and a total of around 400 CAZymes in its genome. But the composition of secretome varies depending on the carbon source used, culture conditions or experimental parameters. The first proteome analysis of *T. reesei* RUT-C30 identified a total of 22 proteins using lactose as carbon source [30]. Another study, using different carbon sources identified 230 extracellular proteins and 90 CAZymes [31]. In the present study, using a minimal mineral salt medium under identical conditions, a total of 53 proteins were identified from *T. reesei* secretome, while *P. janthinellum* secreted 85 different proteins. As expected, most of the proteins identified from both the fungi were related to biomass degradation. More number of CAZymes was identified from *P. janthinellum* secretome. CAZymes from *T. reesei* included 2 cellobiohydrolases, 3 endoglucanases, 9 hemicellulases and the accessory activities—swollenin and lytic polysaccharide monooxygenase (LPMO). CAZymes from *P. janthinellum* were grouped into 12 cellobiohydrolases, 3 endoglucanases, and 6 hemicellulases. No beta-glucosidases were identified from both secretomes to support the extremely higher beta-glucosidase activity shown by *P. janthinellum*. However, the number of cellobiohydrolases and their relative abundance was very high in *P. janthinellum*. The proteins identified from the secretome may not be a complete representation of all the CAZymes secreted by the organism, as the study used only a single time point and pure cellulose as sole carbon source. The highest differentially expressed protein from *T. reesei* was the GH7 family endoglucanase EG-1, which showed 183,567-fold increase in expression upon cellulose induction. However, CBH1 is known to be the major secreted protein of *T. reesei* on cellulose induction [32]. The difference in this study might be a result of the culture conditions and/or the time point of analysis. It could also result from the processing of samples where the insoluble cellulose fraction, which could bind the enzyme, was removed to obtain the supernatant used for analyses. The normalized fold difference shown by the most highly expressed protein from *P. janthinellum* was almost 2 million, and this was a cellobiohydrolase from GH7 family. While *T. reesei* secreted a wider variety of enzymes involved in lignocellulose hydrolysis, it was the *P. janthinellum* that secreted more glycosyl hydrolases and especially very high level of exoglucanases. The study provides preliminary information on the presence of all major cellulolytic and hemicellulolytic activities in the fungus and a very high induction in presence of cellulose, which could account for its enhanced hydrolytic performance.

## Conclusions

Here, we provide the first ever secretome analysis of *Penicillium janthinellum* NCIM1366 and its comparison with the established cellulase hyper producing industrial strain—*Trichoderma reesei* RUT-C30. The analyses have highlighted the better hydrolytic efficiency, enzyme activity, protein production and secretion efficiency of *P. janthinellum,* which indicates its potential as future industrial cellulase producer. Further exploration and a deeper understanding on the reasons of its better cellulase production warrants genome and transcriptome level studies on the fungus which is progressing and we aim to reveal soon. Further targeted genetic modifications are expected to improve its performance even more, providing a worthy alternative for *T. reesei*, or complement it in the cellulase applications for biomass conversion.

## Methods

### Microorganism and growth medium

*Penicillium janthinellum* NCIM1366 was kindly provided by National Culture Collection of Industrial Microorganisms (NCIM), CSIR-National Chemical Laboratory, Pune, India, and *Trichoderma reesei* RUT-C30 culture was a kind gift from Prof George Szakacs, Technical University of Budapest. The cultures were grown and maintained in potato dextrose agar (PDA) slant. For enzyme production, spores were collected from 20-day-old PDA slants of *P. janthinellum* NCIM1366 and 5-day-old slants of *T. reesei* RUT-C30.

### Enzyme production

For enzymatic hydrolysis, cellulase enzyme production was carried out under submerged fermentation using optimized media for both organisms. Both media were modified from the original Mandels and Weber medium [25], optimized for growth and cellulase production of the respective organisms. The enzyme production medium for *P. janthinellum* contained (in g/L): KH_2_PO_4_ (2.0), CaCl_2_.2H_2_O (0.3), urea (0.3), MgSO_4_.7H_2_O (0.3), (NH4)_2_SO_4_ (1.4), peptone (0.75), yeast extract (0.25), Tween-80 (0.5) and trace elements: FeSO_4_.7H_2_O (0.005), MnSO_4_.H_2_O (0.0016), ZnSO_4_.7H_2_O (0.0014), and CoCl_2_.6H_2_O (0.002) with the pH of medium adjusted to 5.5. Cellulose (1% w/v) and wheat bran (2.5% w/v) were used as carbon sources and a spore suspension containing 1 × 10^5^ spores/ml was used as inoculum at 1% (v/v) level. For *T. reesei*, the production medium contained (in g/L): KH_2_PO_4_ (2.0), (NH_4_)_2_HPO_4_ (2.1), yeast extract (2), NaCl (0.5), CaCl_2_.2H_2_O (0.3), urea (0.3), MgSO_4_.7H_2_O (0.3), Tween 80 (0.5) and trace elements: FeSO_4_.7H_2_O (0.005), MnSO_4_.H_2_O (0.0016), ZnSO_4_.7H_2_O (0.0014) and CoCl_2_.6H_2_O (0.002) with pH of the medium adjusted to 7.2. The carbon sources used were 0.1% lactose, 2% cellulose and 1.5% wheat bran and the medium was inoculated at 1% (v/v) level with a 1 × 10^6^ spores/ml spore suspension. Cultivation was carried out at 30 ± 2 °C and 200 rpm agitation. The extracellular crude enzyme from both cultures was collected after 10 days of incubation, and was assayed for total cellulase activity. Enzyme activity was expressed in filter paper units (FPU).

### Comparison of total cellulase activity, endoglucanase activity, and beta-glucosidase activity

Both organisms were cultivated in the basic Mandels and Weber medium [25] (composition in g/L: KH_2_PO_4_ (2.0), CaCl_2_.2H_2_O (0.3), urea (0.3), MgSO_4_.7H_2_O (0.3), (NH4)_2_SO_4_ (1.4), peptone (0.75), yeast extract (0.25), Tween-80 (0.5) and trace elements (g/L): FeSO_4_.7H_2_O (0.005), MnSO_4_.H_2_O (0.0016), ZnSO_4_.7H_2_O (0.0014), and CoCl_2_.6H_2_O (0.002) with pH of the medium adjusted to 5.0. Cellulose (1% w/v) was used as the sole carbon source. The inoculum size used was 1% v/v of a 1 × 10^5^ spores/ml suspension for both the cultures. Cultivation was carried out at 30 ± 2 °C and 200 rpm agitation. Samples were collected from the 2nd day onwards, every 48 h. Total cellulase activity (filter paper activity) and endoglucanase (CMCase) activity was determined by the IUPAC method [33] and beta-glucosidase activity was determined as described by Rajasree et al. [34]. One unit of beta-glucosidase activity is defined as the amount of enzyme required to liberate 1 μg *p*-nitro phenol from pNPG (4-nitrophenyl β-D-glucopyranoside) per milliliter.

### Zymogram analysis of extracellular proteins

Native poly-acrylamide gel electrophoresis (PAGE) of the extracellular enzyme and methyl umbelliferyl β-D-glucopyranoside (MUG) staining was performed as described by Rajasree et al. [34].

### Comparison of hydrolytic efficiencies and cellobiose accumulation

Different lignocellulosic biomass, pretreated similarly using either dilute acid or alkali, was used for the hydrolysis studies. For acid pretreatment, biomass (20% w/v) was mixed with 10% w/w of H_2_SO_4_ and was pretreated for 1 h at 120 ± 2 °C. The biomass was cooled to room temperature and a slurry was made by adding 2 × volume of water. The pH of the slurry was adjusted to 6.0 by adding 10 N NaOH. Solid–liquid separation was performed using a nylon sieve and the biomass was washed twice with tap water. The biomass was used directly after correction of moisture or air dried at room temperature and stored until used. For alkali pretreatment, 20% w/v rice straw and 10% w/w NaOH was mixed and pretreated at 120 ± 2 °C for 1 h. Water (2 × volume) was added and the pH of the pretreated slurry was adjusted to 6 by adding 10 N H_2_SO_4_. The biomass was then processed as described above.

The hydrolysis reactions (20 ml reaction volume) were carried out in screw-capped glass conical flasks with the following conditions: 10% w/w of dry biomass, 10 FPUs/g enzyme loading and 0.05% w/w surfactant (Tween 80) loading. 0.5% v/v of a commercial penicillin/streptomycin mixture (Himedia, India) was added to prevent any bacterial contamination. The hydrolysis reaction was carried out at 50 °C for 24 h and samples were collected at 0, 4, 8, 12 and 24 h of hydrolysis. The amount of glucose, xylose, arabinose, mannose and cellobiose in the samples was determined using the HPLC as described previously [35].

### Comparison of extracellular protein production and secretome analysis

Both organisms were grown in basic Mandels and Weber medium [25] with either glucose or cellulose at 1% (w/v) level as sole carbon sources, as described above. Samples were collected at 48-h intervals starting from the 2nd day. Total secreted proteins present in the samples were estimated by the Bradford method [36] with BSA as standard. SDS-PAGE was carried out as described by Laemmli [37], using 12% acrylamide gels. Proteins were visualized by staining with Coomassie Brilliant Blue R-250.

For secretome analyses, extracellular proteins were collected by centrifugation for 10 min at 4 °C and 13,400 × g after 10 days of incubation. The supernatants were further clarified by filtration through 1μ Glass microfiber filter. The collected proteins were dialyzed against 50 mM ammonium bicarbonate buffer and concentrations were normalized. Samples were digested using trypsin following the standard protocol [38]. The proteomic profiling was performed in duplicates by liquid chromatography tandem mass spectrometry (LC–MS/MS) at the Mass Spectrometry & Proteomics Core facility of Rajiv Gandhi Centre for Biotechnology, Trivandrum, India. The protein samples were subjected to in-solution trypsin digestion using sequence grade trypsin (Sigma Aldrich, India). The LC/MS/MS analyses of the tryptic peptides were performed in a SYNAPT G2 high definition mass spectrometer (Waters, UK), connected to a NanoACQUITY UPLC® chromatographic system (Waters, UK) for the separation of the peptides. The LC–MS/MS acquired raw data were analyzed by Progenesis QI for Proteomics V3.0 (NonLinear Dynamics, Waters, UK) for protein identification using the protein database of *Trichoderma reesei* and *Penicillium* downloaded from UniProt repository. Prediction of the presence of secretion signal motifs was achieved using SignalP 5.0 [39].

### Identification of CAZymes from secretome data

Annotation of CAZy (carbohydrate active enzymes) family of proteins as per CAZy database [40] was done through dbCAN2 meta server [41].

## Supplementary information

**Additional file 1.** Proteins identified from the induced and uninduced secretome of *T. reesei* RUTC30.

**Additional file 2.** Proteins identified from the induced and uninduced secretome of *P. janthinellum* NCIM 1366.

## Data Availability

All essential data generated or analyzed during this study are included in this published article and its supplementary information files. More elaborate datasets generated during and/or analyzed during the current study available from the corresponding author on reasonable request.
